# Prestin Contributes to Membrane Compartmentalization and Is Required for Normal Innervation of Outer Hair Cells

**DOI:** 10.3389/fncel.2018.00211

**Published:** 2018-07-20

**Authors:** Satoe Takahashi, Willy Sun, Yingjie Zhou, Kazuaki Homma, Bechara Kachar, Mary Ann Cheatham, Jing Zheng

**Affiliations:** ^1^Department of Otolaryngology – Head and Neck Surgery, Feinberg School of Medicine, Northwestern University, Chicago, IL, United States; ^2^Section on Structural Cell Biology, National Institute on Deafness and Other Communication Disorders, National Institutes of Health, Bethesda, MD, United States; ^3^Department of Communication Sciences and Disorders, Northwestern University, Evanston, IL, United States; ^4^The Knowles Hearing Center, Northwestern University, Evanston, IL, United States

**Keywords:** prestin, outer hair cells, efferent innervation, PMCA2, KCNQ4

## Abstract

Outer hair cells (OHC) act as amplifiers and their function is modified by medial olivocochlear (MOC) efferents. The unique OHC motor protein, prestin, provides the molecular basis for somatic electromotility, which is required for sensitivity and frequency selectivity, the hallmarks of mammalian hearing. Prestin proteins are the major component of the lateral membrane of mature OHCs, which separates apical and basal domains. To investigate the contribution of prestin to this unique arrangement, we compared the distribution of membrane proteins in OHCs of wildtype (WT) and prestin-knockout (KO) mice. In WT, the apical protein PMCA2 was exclusively localized to the hair bundles, while it was also found at the lateral membrane in KOs. Similarly, a basal protein KCNQ4 did not coalesce at the base of OHCs but was widely dispersed in mice lacking prestin. Since the expression levels of PMCA2 and KCNQ4 remained unchanged in KOs, the data indicate that prestin is required for the normal distribution of apical and basal membrane proteins in OHCs. Since OHC synapses predominate in the basal subnuclear region, we also examined the synaptic architecture in prestin-KO mice. Although neurite densities were not affected, MOC efferent terminals in prestin-KO mice were no longer constrained to the basal pole as in WT. This trend was evident as early as at postnatal day 12. Furthermore, terminals were often enlarged and frequently appeared as singlets when compared to the multiple clusters of individual terminals in WT. This abnormality in MOC synaptic morphology in prestin-KO mice is similar to defects in mice lacking MOC pathway proteins such as α9/α10 nicotinic acetylcholine receptors and BK channels, indicating a role for prestin in the proper establishment of MOC synapses. To investigate the contribution of prestin’s electromotility, we also examined OHCs from a mouse model that expresses non-functional prestin (499-prestin). We found no changes in PMCA2 localization and MOC synaptic morphology in OHCs from 499-prestin mice. Taken together, these results indicate that prestin, independent of its motile function, plays an important structural role in membrane compartmentalization, which is required for the formation of normal efferent-OHC synapses in mature OHCs.

## Introduction

Outer hair cells (OHC) are unique cells that are capable of performing somatic length changes (Brownell et al., 1985) to enhance mechanical displacements of the organ of Corti. Therefore, OHCs in the inner ear function as cochlear amplifiers (Davis, 1983), and are required for high sensitivity and sharp frequency selectivity in mammals (Dallos, 1992). OHC somatic electromotility is mediated by the motor protein, prestin (Zheng et al., 2000), which belongs to a diverse anion transporter family called solute carrier protein 26 (SLC26). Unlike other members of this family, prestin undergoes a voltage-dependent conformational change to confer electromotility (Zheng et al., 2000; Dallos et al., 2008). Prestin-knockout (KO) mice exhibit a complete loss of OHC electromotility resulting in ∼50 dB threshold shift, and loss of frequency selectivity (Liberman et al., 2002; Cheatham et al., 2004). Therefore, prestin is essential for mammalian cochlear amplification.

The lateral membrane (LM) of wildtype (WT)-OHCs is packed with ∼11 nm particles presumably containing prestin tetramers (Zheng et al., 2006; Kumano et al., 2010; Wang et al., 2010; Hallworth and Nichols, 2012). It is, therefore, understandable that OHCs without prestin are ∼40% shorter than normal (Wu et al., 2004; Cheatham et al., 2007, 2015). Because Dieters’ cells elongate to compensate for the shorter OHCs, the general anatomical structure of organ of Corti appears normal (Liberman et al., 2002). Surprisingly, prestin-KO mice also suffer from premature OHC death (Wu et al., 2004; Cheatham et al., 2007, 2015), suggesting that prestin may be involved in OHC maintenance and survival in addition to its role in electromotility. Yamashita and colleagues also reported that proteins in the OHC’s LM, including prestin, are virtually immobile (Yamashita et al., 2015), suggesting that the prestin-embedded LM forms a distinct domain, separate from apical and basal membrane compartments. This unique arrangement of membrane domains appears to be functionally important, as hypothyroid animals, where prestin expression is observed along the entire basolateral membrane, have impaired hearing and reduced electromechanical force generation (Cimerman et al., 2013). In this regard, prestin is the key intrinsic factor that separates motile OHCs from non-motile inner hair cells (IHC), which only have two (apical and basolateral) membrane compartments.

**FIGURE 1:**
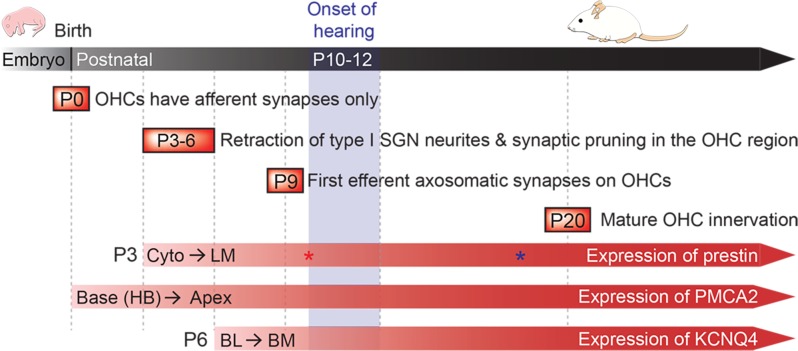
Postnatal development of the cochlea. Developmental milestones are indicated for both afferent and efferent synapses. Prestin protein expression begins around P3 in cytoplasmic vesicles (Cyto), and then moves exclusively to the lateral membrane (LM) (Legendre et al., 2008). Prestin protein expression levels reach a maximum by P10 (shown with red asterisk), while electromotility (NLC) matures by P18 (shown with blue asterisk) (Abe et al., 2007). PMCA2 protein is observed at P0 in hair bundles (HB) at the base of cochlea, and then eventually reaches the apex by P6 (Chen et al., 2012). KCNQ4 proteins are found at the basolateral (BL) membrane by P6, and then quickly localizes to the basal membrane (BM) (Winter et al., 2006). The onset of hearing in mice is around P10–12 (modified from Bulankina and Moser, 2012).

The literature indicates that the onset of hearing in rodents occurs at 10–12 days of life (Bulankina and Moser, 2012), which overlaps with the expression of prestin protein and the emergence of OHC electromotility (He et al., 1994; Belyantseva et al., 2000; Abe et al., 2007) (**Figure 1**). During this period, many membrane proteins are also expressed and targeted to their final destinations. For example, plasma membrane Ca^2+^ ATPase (PMCA2) proteins first appear at the stereocilia/apical membrane (AM) of OHCs located at the basal turn of the cochlea at ∼P0, and then gradually progress to apical locations at P6 (Dumont et al., 2001; Chen et al., 2012) (**Figure 1**). Potassium voltage-gated channel subfamily KQT member 4 (KCNQ4) is expressed in the entire basolateral membrane of OHCs, similar to prestin in the first few days after birth (P6). Subsequently, KCNQ4 proteins shift to the basal pole after the onset of hearing as prestin proteins become restricted to the LM (P8–P12) (Boettger et al., 2002; Winter et al., 2006). Around the time when prestin protein leaves the basal area, synapses between OHCs and medial olivocochlear (MOC) efferents are formed (P9). In adult mice, most efferent-OHC synapses are found at the basal membrane (BM) of OHCs. Acetylcholine (ACh) released from MOC presynaptic terminals activates α9/α10 nicotinic ACh receptors (nAChR) located at the BM, and ultimately induces outward potassium currents via SK2 and BK potassium channels. The resulting hyperpolarization is thought to reduce cochlear gain, by attenuating OHC electromotility (Fuchs, 2014; Guinan, 2014). This efferent feedback system is thought to improve signal selection in noisy environments, and to protect against noise-induced trauma and aged-related hearing loss (Maison et al., 2013b). Therefore, mature OHCs have three distinct domains with distinct functions: AM for receiving mechanical inputs (transducer channels); LM for somatic electromotility (prestin); and BM for neural communications (synapses with MOC efferents and type II afferents).

Establishing the LM domain as a separate compartment appears to be unique to OHCs. As prestin is abundantly expressed only in the LM of OHCs, we hypothesize that prestin is one of the key factors inducing this arrangement. To demonstrate prestin’s role in OHC membrane compartmentalization, we first examined cochlear samples from prestin WT mice and prestin-KO mice that lack prestin protein (Liberman et al., 2002). PMCA2 is used as an apical protein marker, while KCNQ4 is used as a basolateral membrane protein marker. Our data show that PMCA2 and KCNQ4 are widely dispersed at both apical and lateral membranes in prestin null mice (KO) even though the expression of PMCA2 protein and KCNQ4 mRNA are not changed in prestin-KO mice. These data suggest that the prestin-embedded LM is needed to establish normal distributions of apical, lateral, and basal membrane proteins. Since most OHC synapses are formed at the base of OHCs, we further investigated synaptic formation in prestin-KO mice at different developmental ages. Although the development of afferent and efferent innervation seems independent of the presence of prestin, MOC efferent terminals in prestin-KOs resemble the phenotypes observed in KO mouse models for α9/α10 nicotinic ACh receptors and BK channels. Thus, in the absence of prestin protein, MOC pathway proteins such as α9, α10, and BK, may fail to form mature synapses as the barrier that restricts their localization to the basal membrane does not exist, resulting in diffused distribution of the terminals. To investigate whether prestin’s motile function contributes to the establishment of OHC LM domains, a prestin-knockin (KI) mouse model that expresses virtually non-electromotile prestin 499-prestin (Dallos et al., 2008) was used. We found that PMCA2 localization was restricted in the hair bundles of OHCs in 499-prestin-KI as in WT, and 499-prestin-KI mice also had normal OHC-MOC synapses. Collectively, our data suggest that prestin, independent to its motile function, plays an important structural role in establishing the lateral and basal membrane boundaries required for the proper formation of OHC-MOC efferent synapses.

## Materials and Methods

### Animals

All experimental procedures were conducted in accordance with the Guide for the Care and Use of Laboratory Animals by NIH and were approved by Northwestern University’s Institutional Animal Care and Use Committee (IACUC), and by the National Institute on Deafness and Other Communication Disorders Animal Care and Use Committee (NIDCD ACUC, protocol #1215). Prestin WT, homozygote (KO), and homozygote 499-prestin (V499G/Y501H) knockin (KI) mice were used in this study. Prestin KO and 499-prestin-KI mice on the FVB background were generated by backcrossing the original mouse models (129/C57BL/6 background (Liberman et al., 2002; Dallos et al., 2008; Cheatham et al., 2015) to the FVB strain for 8 generations and then maintaining them for several years without refreshing the background strain. All animals were maintained by both homotypic and heterotypic breeding, and genotypes were determined by Transnetyx (Cordova, TN, United States). Both males and females are used in all experiments.

### Tissue Processing and Immunofluorescence

Mice were cardiac perfused with 4% paraformaldehyde and cochleae extracted, post-fixed in 4% paraformaldehyde for 2 h at room temperature or overnight at 4°C, and decalcified in 0.12M EDTA in 1X PBS for 2 days at room temperature. For whole-mount preparations, cochleae were dissected following the Eaton-Peabody Laboratory cochlear dissection protocol (Liberman et al., 2015). For radial sections, decalcified cochleae were placed in a series of sucrose solutions in 1X PBS (10–30%) and embedded in OCT, sectioned into 12-micron slices using a Microm HM 505N cryostat, and placed on glass slides. Samples were then blocked (1X TBS containing 5% normal goat serum and 0.1–1% Triton X-100) and incubated with primary antibodies at 4°C for prestin (Zheng et al., 2005) and KCNQ4 (Beisel et al., 2005), at room temperature for PMCA2 (Thermo, **RRID**: AB_2243199), or 37°C for NF-200 (Millipore, **RRID**: AB_177520), Tubb3 (BioLegend, **RRID**: AB_231377), Synaptophysin (Millipore, **RRID**: AB_11214133), VAChT (Millipore, **RRID**: AB_2630394) in a humid chamber overnight. Samples were then washed in 1X PBS and incubated with appropriate fluorophore-conjugated secondary antibodies, including goat anti-rabbit (Thermo, **RRID**: AB_10374301), anti-mouse Alexa Fluor 488 (**RRID**: AB_2556548) or 546 (Thermo, **RRID**: AB_2534103), and Fluorescein goat anti-chicken IgY (Aves Labs, **RRID**: AB_2313516), for at least 2 h at room temperature or 37°C in a humid chamber. Alexa 546-conjugated phalloidin (Thermo, A22283) and Hoechst 33342 (Thermo, H3570) were also included to stain actin and nuclei, respectively (Takahashi et al., 2016). Stained cochlear sections were mounted onto slides using Dako fluorescent mounting medium (Agilent), and imaged using Nikon C2 or A1R+ confocal microscopy. Z-stacks were captured using step sizes of 0.5 μm. Images were analyzed using Image J, Nikon NIS Element, and Imaris 8 (Bitplane) software. Statistical analyses were carried out using Prism 7 (GraphPad) software.

### Transmission Electron Microscopy

Cochlear samples from WT and prestin-KO mice were prepared for electron microscopy as previously described (He et al., 2010). Briefly, microdissected cochleae were fixed with 4% paraformaldehyde and 0.5% glutaraldehyde, plunge frozen, and transferred to a Leica Biosystems AFS for freeze substitution. Tissues were submerged in 1.5% uranyl acetate in absolute methanol at -90°C for 2 days and then infiltrated with HM20 Lowicryl resin (Electron Microscopy Sciences) over 2 days at -45°C. Resin was polymerized under UV light with temperatures rising from -45 to 0°C across 3 days. Ultrathin sections were produced using a Leica ultramicrotome, collected onto 300-mesh hexagonal Ni grids (Electron Microscopy Sciences), and examined using a 200 kV JEOL 2100 electron microscope equipped with a Gatan Orius 832 CCD camera. Images were acquired with DigitalMicrograph (Gatan). Fiji software was used to crop, rotate and adjust image brightness and contrast.

### Isolation of OHCs for Single Cell RNA Sequencing

Outer hair cells were collected from male WT, prestin-KO and 499-KI mice as described before (He et al., 2000; Cheatham et al., 2005). Briefly, cochleae were dissected at P28 and placed in PBS (pH 7.4, 339 mOsm). After removing the bony cochlear wall, the organ of Corti from the apical turn was carefully removed and treated with 1 mg/ml type IV Collagenase (Sigma) in L-15 for 6 min. After removing the enzymatic solution, fresh PBS was added to the sample tube. Gentle pipetting allowed individual OHCs to be separated from the cellular matrix. The samples were then placed in a chamber mounted onto the stage of an inverted microscope (Leica DM IRB). Two solitary OHCs were collected using a pipette pulled from a borosilicate glass capillary (tip diameter of 50–60 mm). To ensure only OHCs were collected for RNA sequencing, the OHCs were placed on a glass slide in a droplet containing 5 ml PBS and inspected for their morphology. OHCs were mixed with Reaction Buffer (total 10.5 ml) provided in the SMART-Seq V4 Ultra Low Input RNA Kit (Clontech). All OHCs were collected within 1 h after the mice were euthanized and stored in the Reaction Buffer at -80°C until processed. OHC single cell RNA sequencing and basic bioinformatics analysis was performed by the NUSeq Core facility at Northwestern University. Full-length, double stranded cDNA was amplified 20 cycles to obtain enough material for RNA sequencing using Illumina NextSeq 500.

### RT-PCR Analysis of Chrna9 Transcripts

Total RNA from cochlear samples was prepared immediately after euthanasia by directly placing the extracted cochleae into lysis buffer, and processing the samples using the Absolutely RNA Miniprep Kit (Agilent). 0.5 μg total RNA was used for cDNA synthesis using Transcriptor Reverse Transcriptase (Roche). *Chrna9* mRNA was detected by PCR as previously described (He et al., 2001). The *Chrna9* primer pairs (sense: 5′-TCTACATCGTCAACCTCCTCATC, antisense: 5′-CTTCGTGACTTGTTCTGGCTCCT) were used to amplify a 400 bp fragment from the cDNA. Cyclophilin primers (sense; 59-TGGCACAGGAGGAAAGAGCATC-39; antisense; 59-AAAGGGCTTCTCCACCTCGATC-39) that amplify a 301-bp DNA fragment were used as an internal control. Cycle parameters were 30 seconds (s) at 95°C followed by 45 cycles of 95°C for 20 s, 55°C for 45 s and 68°C for 45 s.

### Protein Analysis Using Western Blotting

Cochleae were dissected from WT and prestin-KO mice around 1 month of age. Paired cochlea from each mouse were lysed in CelLytic MT Cell Lysis Reagent (Sigma) supplemented with 1X protease inhibitor cocktail (Sigma), 1 mM PMSF, and 10 μg/ml DNase I. Protein concentrations in the lysates were measured using the Thermo 660 nm protein assay reagent. Thirty microgram of total lysates were loaded per lane of a 4–20% gradient gel, and PMCA2 detected by anti-PMCA2 (1:2000), followed by goat anti-rabbit IgG-HRP (1:5000). For the loading control, Tubulin was detected by anti-α-Tubulin (Zymed laboratories Inc., 1:1000) and goat anti-mouse HPR (1:5000). Signals were detected using a SuperSignal West Pico Chemiluminescent Substrate (Thermo). A Kodak Imaging System was used to capture the images. Band intensities of PMCA2 and Tubulin were measured using ImageJ, and plotted using Prism 7 (GraphPad). The Welch’s unequal variances *t*-test was also used to compare mean PMCA2 band intensities of prestin-WT and KO mice after normalizing to Tubulin, the housekeeping control.

## Results

### Ectopic Localization of Hair Bundle Protein PMCA2 in OHCs From Prestin-KO but Not in 499-Prestin-KI

**FIGURE 2:**
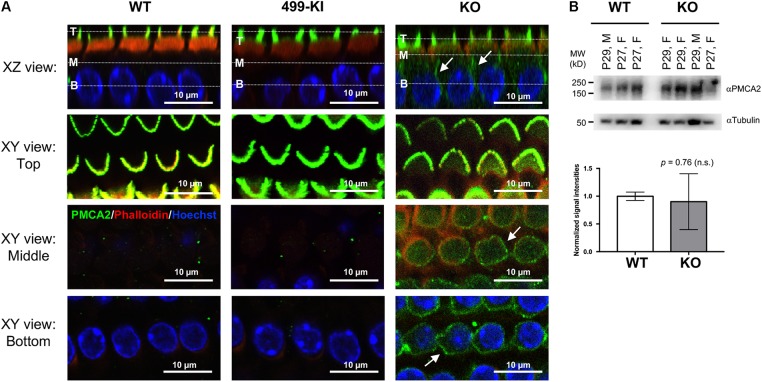
PMCA2 expression in OHCs. **(A)** Representative images of OHCs from prestin-WT, 499-prestin-KI, and prestin-KO showing PMCA2 expression in the hair bundles (all) and in the LM of OHC (KO only). Cochlear whole mounts from the middle turn of WT (P12, male), 499-KI (P12, female) and prestin-KO (P27, male) mice were stained with anti-PMCA2 (green). 3D reconstructions of z-stack images show radial views (XZ) of OHCs. White dotted lines in the XZ views indicate the Z-positions of XY views at the top (T), middle (M), and the base (B) of the OHC. PMCA2 was found exclusively at the hair bundles of OHCs in WT and 499-KI mice. In prestin-KO, PMCA2 signals were also present along the lateral walls (arrows, XZ and XY views). Scale bars, 10 μm. **(B)** Representative images of Western blots showing PMCA2 proteins detected in cochlear samples of WT (*n* = 3) and prestin-KO (*n* = 4) mice. Tubulin protein is used as loading control. Normalized intensities of PMCA2 (intensity of PMCA2/intensity of tubulin) in WT and prestin-KO cochleae are plotted (mean ± SD), and no significant difference was found (Welch’s unequal variances *t*-test).

Apical proteins are transported to the apical membrane by either direct trafficking from the *trans*-Golgi network (Keller and Simons, 1997), or by an indirect transcytotic route. In the latter process, proteins are first delivered to the basolateral membrane before being internalized and then transcytosed to the apical membrane (Mostov et al., 1999; Polishchuk et al., 2004). Both pathways can be used in the same cell and the routing of apical proteins can even switch from one pathway to the other during epithelial polarity development (Zurzolo et al., 1992; Weisz and Rodriguez-Boulan, 2009). For mature OHCs, PMCA2 was found exclusively at the hair bundles in WT (Hill et al., 2006; Chen et al., 2012) (**Figure 2A**, *n* = 7, P12–94). Although PMCA2 was found in the hair bundles of prestin-KOs, it was also detected in the LM (**Figure 2A**, *n* = 4, P20–35). This result is consistent with that of Hill and colleagues who noted imperfect targeting of PMCA2 proteins to the basolateral membrane in immature OHCs (P2) before prestin protein is expressed, as well as when PMCA2 is expressed using a gene-gun transfection method (Hill et al., 2006). Thus, in the absence of prestin, apical targeting of PMCA2 is less effective as in immature WT-OHCs, which may be due to stalling along the transcytotic pathway. As the expression of PMCA2 is reported to increase after the onset of hearing (Watson et al., 2014), we examined PMCA2 protein levels in WT (*n* = 6) and prestin-KO (*n* = 7) cochleae by Western blot. As shown in **Figure 2B**, there are no significant differences in PMCA2 expression levels in WT and prestin-KO cochleae, suggesting the prestin-KO mice retain mature levels of PMCA2 protein.

Outer hair cells lacking prestin are shorter than WT. To eliminate potentially deleterious effects due to the anatomical changes in prestin-KO, the 499-KI mouse model was created, which expresses prestin V499G/Y501H with proper targeting to the LM of OHCs (Dallos et al., 2008; Homma et al., 2013, Supplementary Figure S2). Although 499-OHCs retain normal length, axial stiffness, and forward transduction, 499-KI mice do not have electromotility at normal OHC resting potentials or at high frequencies (Dallos et al., 2008; Homma et al., 2013). Interestingly, we did not observe ectopic localization of PMCA2 in OHCs from 499-prestin-KI mice (*n* = 3, P12–18), which expresses virtually non-motile prestin mutant (**Figure 2A**). These data suggest that the presence of prestin protein, regardless of its motile function, influences the apical targeting of PMCA2.

### Ectopic Localization of Basolateral Membrane Protein KCNQ4 in OHCs From Prestin-KO

**FIGURE 3:**
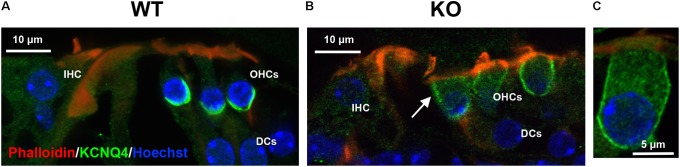
Representative immunofluorescent images of the organ of Corti from prestin WT **(A)** and KO **(B)** mice. **(C)** An enlarged OHC from an apical turn of prestin-KO cochlea (P21, male). Radial cochlear sections from WT (P138, male) and prestin-KO (P37, male) mice were stained with anti-KCNQ4 (green). Phalloidin-Alexa 546 (red), and Hoechst 33342 (blue) for actin and nuclei, respectively, were also used. White arrow indicates KCNQ4 signal on the LM of OHC in prestin-KO mouse. IHC, inner hair cells; DCs, Dieters’ cells. Scale bars, 10 μm.

Next, we examined the localization of KCNQ4, a basal membrane protein, in prestin-KO OHCs. KCNQ4 is localized throughout the entire basolateral membrane at P4–P7 (Boettger et al., 2002; Winter et al., 2006) and coalesces at the basal pole as OHCs mature (**Figure 1**). KCNQ4 is concentrated only at the base of mature OHCs in prestin-WT (**Figure 3A**, *n* = 3, P49–194) as previously reported. However, in OHCs from prestin-KO mice, KCNQ4 is found along the entire basolateral membranes, reminiscent of its immature state (**Figures 3B,C**, *n* = 3, P21–37). We also isolated OHCs from WT and prestin-KO, and performed single cell RNA sequencing (ScRNA-seq, *n* = 3). Differential gene expression of KCNQ4 messages revealed no statistical differences between WT and prestin-KO (data not shown). These data suggest that the absence of prestin protein disorganizes the distribution patterns of basolateral membrane proteins.

### MOC Synapses Are Disorganized in Prestin-KO Mice, but Not in 499-Prestin-KI

**FIGURE 4:**
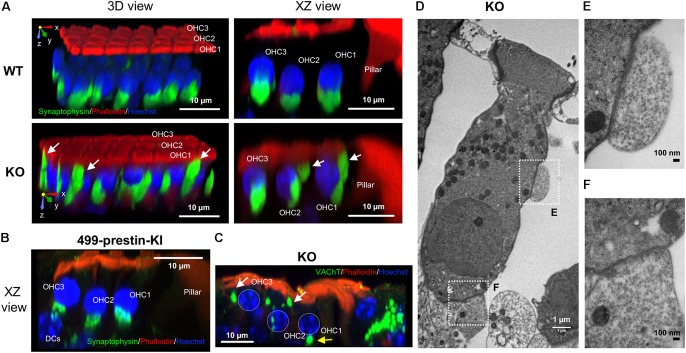
Abnormal localization of MOC synapses in OHCs from prestin-KO mice. Representative images are shown for each panel. **(A)** 3D reconstructions of z-stack images of cochlear whole mounts from the middle turn of 129/B6 WT (P55, female) and prestin-KO (P26, male) mice, showing three rows of OHCs oriented such that the cuticular plate is facing upward and the first OHC row is toward the front. In WT, MOC terminals are found at the bottom of the OHCs, while in the prestin-KO, MOC terminals are disorganized and extend upward to the cuticular plate. Green: anti-synaptophysin for synaptic vesicles. Red: phalloidin-Alexa 546 for actin. Blue: Hoechst 33342 for nuclei. **(B)** 3D reconstructions of z-stack images of cochlear whole mounts from the middle turn of 499-prestin-KI (P21, female) mouse, showing three rows of OHCs. MOC terminals (stained with anti-synaptophysin) were found at the bottom of the OHCs. **(C)** 3D reconstruction of z-stack images of a cochlear whole mount from the apical turn of a prestin-KO (P22, male), stained with anti-VAChT (green), phalloidin-Alexa 546 (actin) and Hoechst 33342 (nuclei; OHC nuclei were marked with white dotted circles). MOC terminals above OHC nucleus (white arrows) are often present in addition to the subnuclear terminals at the bottom (yellow arrow). Scale bar, 10 μm. **(D)** TEM image of prestin-KO OHC with two MOC terminals at the side **(E)** and the bottom **(F)** of the cell. Boxed regions correspond to the images in **(E,F)**, respectively. 1200×, scale bar, 1 μm. **(E,F)** Enlarged images of supranuclear synapse along the LM of OHCs **(E)** and subnuclear synapse **(F)**. Both terminals in **(E,F)** contain numerous small synaptic vesicles. 10,000×, scale bars, 100 nm.

In humans, MOC efferents innervating OHCs form two kinds of synapses that are separated by the prestin-embedded LM: subnuclear and supranuclear synapses (Nadol and Burgess, 1994). Supranuclear synapses are located near the cuticular plate and are rarely observed or studied due to their reduced size/incidence. In contrast, anatomical studies have shown that the subnuclear synapses at the basal pole of OHCs contain acethylcholine (ACh) and other neurotransmitters associated with MOC efferents (Guinan, 1996). Since the distribution of apical and basal membrane proteins becomes less constrained in the absence of prestin, we sought to investigate whether the lack of prestin in the LM also affects OHC-MOC synapses. Cochlear samples from adult WT and prestin-KO mice were stained with anti-synaptophysin, a marker for synaptic vesicles. We examined the distribution of synaptophysin signals and found prominent subnuclear synapses and fewer/smaller supranuclear synapses near the cuticular plate in WT cochleae (*n* = 8, P27–55) similar to that reported in humans (Supplementary Figure S1). In prestin-KO mice, however, the synaptophysin signals were widely distributed along the LM of OHCs in addition to the supranuclear and subnuclear places (**Figure 4A** and Supplementary Figure S1, *n* = 5, P20–41). To investigate whether prestin’s motile function contributes to the disorganized MOC synapses, we also examined the MOC synapses in OHCs from 499-presitn-KI mice. As shown in **Figure 4B** (*n* = 4, P18–32), synaptophysin signals were prominently associated with subnuclear synapses, similar to the expression patterns observed in OHCs from WT mice shown in **Figure 4A**. Compared to WT and 499-KI, the MOC supranuclear terminals in prestin-KO mice were larger, and more numerous (**Figure 4A** and Supplementary Figure S1). By using an antibody against vesicular acetylcholine transporter (VAChT), which is specific to cholinergic efferent terminals, we also confirmed that enlarged supranuclear and subnuclear synapses were VAChT positive (**Figure 4C**, KO, *n* = 3, P20–22). Thus, MOC terminals in prestin-KOs were not constrained to the basal end of the OHCs as in WT and 499-KI. In addition, transmission electron microscopy (TEM) in prestin-KO OHCs found synaptic terminals on the sides of cells near the cuticular plate in addition to those at the bottom of the cell (**Figures 4D–F**, *n* = 4). Both enlarged supranuclear and subnuclear terminals contained dense vesicles in prestin-KO mice, suggesting that these structures might represent functional efferent terminals.

### Disorganization of MOC Terminals in Prestin-KO Mice Is Evident at the Onset of Hearing

**FIGURE 5:**
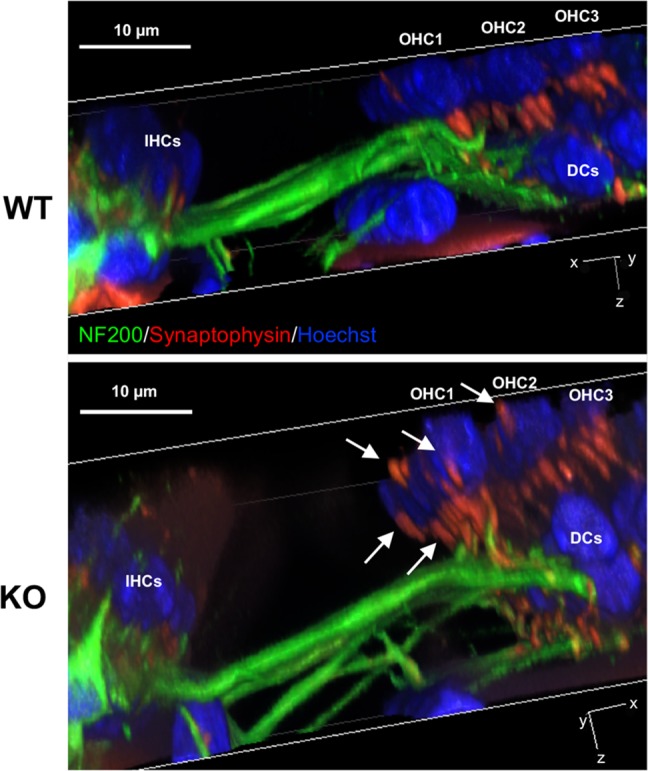
Medial olivocochlear terminals are mislocalized at P12 in prestin-KO cochleae. Representative images of 3D-reconstructed z-stacks are shown for P12 female WT and prestin-KO mice. Cochlear whole mounts were stained for MOC terminals (red, synaptophysin) and neurofilaments (green, NF200). In contrast to WT, white arrows indicate both sub and supranuclear MOC terminals in mice lacking prestin. IHC, inner hair cells; DC, Dieters’ cells. Blue, Hoechst. Scale bar, 10 μm.

Prestin expression starts between P3–5 and progresses from base to apex. Initially, prestin is located in the cytoplasm of OHCs with minimal localization at the PM (**Figure 1**). At this stage, no synapses are found between OHCs and MOC terminals (data not shown). From P7 to P12, prestin protein is redistributed to the lateral membrane (Belyantseva et al., 2000; Weber et al., 2002), and MOC synapses begin to form. To investigate the impact of prestin on normal efferent-OHC synaptogenesis, we examined OHC synapses at the onset of hearing. At P12–13, most synaptophysin signals coalesce at the bottom of the OHCs in WT and prestin KO mice (**Figure 5**, WT, *n* = 5; KO, *n* = 4) However, in mice lacking prestin staining is also apparent along the sides of the OHCs above the nuclei (indicated by white arrows). These data suggest that restriction of OHC-MOC contacts to the bottom of the cell depends on prestin’s presence in the LM.

**FIGURE 6:**
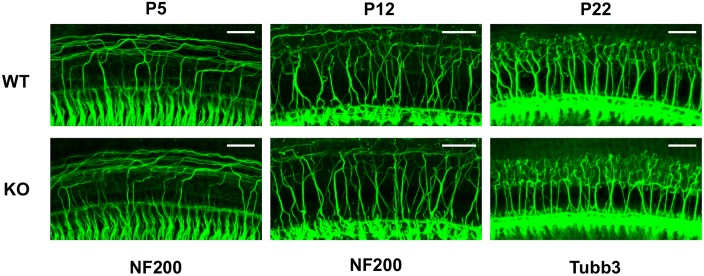
Neuronal trajectories are qualitatively similar in WT and prestin-KO cochleae. Representative images are provided for cochlear whole mounts from WT and prestin-KO mice stained with anti-NF200 (P5 and P12) or anti-Tubb3 (P22). Although it was not possible to determine sex at P5, the P12 samples were from females, and P22 samples were from males. Scale bars, 25 μm.

We also examined the nerve fibers (afferents and efferents) that contact OHCs at P5 (WT, *n* = 4; KO, *n* = 4), P12 (WT, *n* = 3; KO, *n* = 3) and in adult cochleae (WT, *n* = 6, P22–55; KO, *n* = 5, P22–41). We examined cochlear middle turns where the MOC innervation to OHCs is high (Maison et al., 2003). As shown in **Figure 6**, distribution of all fibers (detected by NF200 and Tubb3) in both WT and prestin-KO cochleae was qualitatively similar despite the differences in synaptic locations in prestin-KOs (**Figures 4**, **5**). These data suggest that the trajectories of innervating afferent and efferent fibers to OHCs are independent of the presence of prestin.

### Decreased Numbers of Clusters at MOC Terminals in Prestin-KO Mice

**FIGURE 7:**
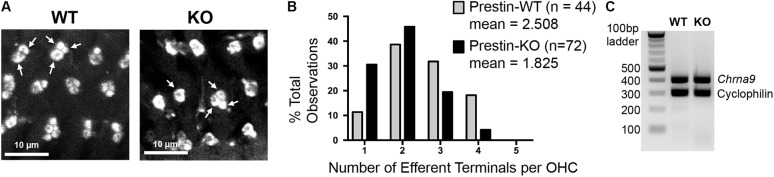
Medial olivocochlear terminals are altered in mice lacking prestin. **(A)** Cochlear whole mounts from the middle turn of prestin-WT and KO mice were stained with anti-synaptophysin. Representative single-slice images of subnuclear synapses from a z-stack series are shown (WT, P27, male; KO, P22, female). In prestin-WT mice, subnuclear MOC terminals were often found in clusters of 2–4 (arrows indicate clusters of 2 and 3), while in prestin-KOs the terminals were more often in singlets or doublets (arrows indicate clusters of 1 and 3). Scale bars, 10 μm. **(B)** Distribution of the MOC terminal clusters between WT and KO was significantly different (*p* = 0.0007, Welch’s unequal variances *t*-test). **(C)** Alpha 9 nAChR expression in prestin-KO mice. RT-PCR using total RNA isolated from prestin-WT (P21, female) and KO (P23, male) cochleae. Cyclophilin was used as an internal control. Prestin-KO showed comparable levels of *Chrna9* mRNA compared to WT.

ACh released from MOC terminals binds to α9/α10 nicotinic ACh receptors on OHCs, leading to the activation of BK channels in the synaptic region. Deletion of these molecular components of MOC feedback loop resulted in hypertrophy and reduced numbers of clusters at MOC terminals in mouse models (Vetter et al., 1999, 2007; Murthy et al., 2009; Maison et al., 2013a). Interestingly, the MOC terminals in prestin-KO mice exhibited abnormal morphology similar to that observed for MOC pathway null mice: they are often enlarged and found in singlets or doublets as compared to the clusters of two or more in WT (**Figures 7A,B**). To confirm this observation, terminals per OHC were counted and compared between WT and prestin-KO mice using confocal z-stack images showing synaptic structures below the midpoint of OHC nuclei. Because MOC terminals are spatially disorganized in prestin-KO mice, this spatial restriction excludes supranuclear clusters. Two sets of WT and KO pairs were analyzed using NIS element software. The raw counts exhibited statistically significant differences (Welch’s unequal variances *t*-test) in the distribution of the MOC terminal clusters (mean numbers of clusters of WT = 2.508 vs. KO = 1,825, *p* = 0.0007). The similarity of abnormal MOC terminal phenotypes of prestin-KO mice and MOC pathway null mice indicates that defects in prestin-KOs may be due to the mislocalization of MOC pathway components due to the absence of prestin protein. We compared the mRNA expression profiles of prestin-WT and KO OHCs using ScRNA-seq (*n* = 3), and found no significant changes for *Chrna9* (α9*), Chrna10* (α10), and *Kcnma1* (BK channels) (Data not shown). Consistently, α9 messages were still detected at comparable levels in prestin-KO cochleae as shown in **Figure 7C** (WT, *n* = 2; KO, *n* = 3). Thus, it is likely that the defects found in MOC terminals in prestin-KO mice are independent of the expression status of MOC pathway components. Collectively, our results suggest that the presence of prestin protein is important for establishing membrane compartments, which in turn direct the formation of normal OHC-MOC synapses.

## Discussion

**FIGURE 8:**
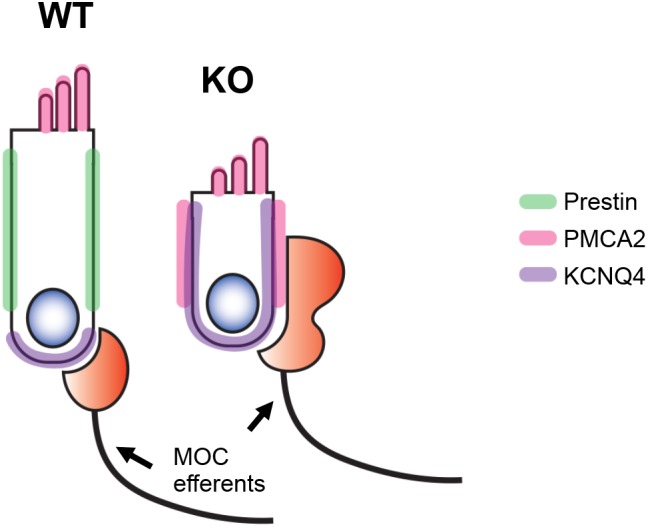
Schematic representation of our results. In the absence of prestin (green), the OHCs fail to develop specialized lateral membrane (LM) domains. As a result, targeting of membrane proteins such as PMCA2 (apical, pink) and KCNQ4 (basal, purple) becomes less restricted. Although the MOC terminals are found at the basal end of OHCs in WT mice, they are mislocalized in prestin-KO mice and become widely distributed along the LM.

In this study, we investigated the role of prestin in establishing proper membrane domains in OHCs. Our experimental evidence in mice indicates that the distribution of apical membrane protein PMCA2 and of basal protein KCNQ4 is not restricted to designated membrane domains in prestin-KO OHCs as it is in WT controls (**Figure 8**). Since PMCA2 can be detected in the LM of immature hair cells (P2) and when newly synthesized from exogenous DNA (Hill et al., 2006), PMCA2 localization at the LM of prestin-KO OHCs may reflect the immature status of OHCs in the absence of prestin. Similarly, KCNQ4 is initially found in the basolateral membrane during neonatal development before coalescing at the bottom of the cell (Boettger et al., 2002; Winter et al., 2006). Hence, the wide distribution of KCNQ4 along the basolateral membrane in prestin-KOs (**Figure 3**) suggests that OHCs never reach their mature status. It is, therefore, conceivable that the postnatal maturation of OHCs depends on prestin expression to attain specialized LM structures.

Outer hair cells are highly specialized cells with a unique trilaminar structure consisting of the subsurface cisternae (SSC), the cortical lattice (CL), and the PM filled with prestin proteins. When OHCs lack prestin, the CL becomes disorganized (He et al., 2010). Since prestin indirectly interacts with one of the cytoskeletal components, βV spectrin (Legendre et al., 2008), which localizes to the lateral wall (Cortese et al., 2017), prestin may be essential for CL organization. In addition, prestin proteins appear to have less mobility in the plasma membrane compared to other membrane proteins. As demonstrated in fluorescence recovery after photobleaching (FRAP) experiments, prestin-GFP expressed in HEK293 cells has a lower effective diffusion coefficient and slower recovery time than other proteins (Organ and Raphael, 2007). Prestin also interacts with cholesterol (Takahashi et al., 2016), which likely contributes to the immobility of the LM domain. In fact, mobility of membrane proteins was observed only when cyclodextrin, a cholesterol chelator, and salicylate, a prestin inhibitor which also vesiculates SSC, were co-applied (Yamashita et al., 2015). These observations indicate that prestin plays an essential role in establishing the specialized LM.

Our experimental results also show mislocalization of MOC terminals in the absence of prestin (**Figure 4**). MOC efferents begin to contact OHCs around the time when prestin expression in the LM nears its maximum (**Figure 1**). Although the initial formation of synapses between hair cells and auditory nerve fibers seems to be largely activity-independent (Bulankina and Moser, 2012; Kersigo and Fritzsch, 2015), the maturation of synapses, as well as the pruning and refinement of cochlear neural networks appears to require inputs from hair cells, auditory neurons, and supporting cells (SC) (Bulankina and Moser, 2012; Knipper et al., 2015). For IHCs, inputs are required from inner SCs (ATP, K+ efflux) in order to establish IHC synaptic patterns (Wang et al., 2015). Although we do not fully understand what makes OHCs expel type I afferents, while retaining type II afferents and MOC contacts during early development, several proteins are known to be important for the formation of OHC-MOC synapses. For example, absence of the postsynaptic ACh receptor complex and the BK channels produces abnormal MOC terminals (Vetter et al., 1999, 2007; Murthy et al., 2009; Maison et al., 2013a), similar to phenotypes observed in prestin-KOs (**Figure 7**). Because the prestin-embedded LM separates apical and basal domains, this arrangement is likely important for the segregation of “cue” proteins at the proper membrane positions to allow normal OHC innervation. In the absence of LM specification due to lack of prestin, it is possible that proteins needed to establish OHC-MOC synapses, such as α9/α10 nAChR, are not spatially restricted to the bottom of the OHCs but instead are widely distributed along the entire basolateral membrane, similar to the expression pattern of KCNQ4 in KO-OHCs (**Figures 3**, **8**). The reduction in MOC terminal clusters in prestin-KO mice (**Figure 7**), which is similar to nAChR and BK KOs (Vetter et al., 1999, 2007; Murthy et al., 2009; Maison et al., 2013a), supports the notion that these proteins might be affected in prestin-KO mice. Although the mRNA expression of these MOC pathway components is unchanged in prestin-KO mice, it is still possible that nAChR and BK channel proteins fail to localize to the basal area to form synapses. Since no good antibodies are available to directly detect nAChR proteins in OHCs, further study is needed to confirm the distribution of these postsynaptic components.

Transmission electron microscopy images showed numerous vesicles and electron dense materials at the membrane where the MOC terminals contact OHCs (**Figures 4D–F**). This observation indicates that the misplaced MOC terminals might represent functional synapses. Although it was speculated that supranuclear synapses in humans might modify the micromechanics (Nadol and Burgess, 1994), this hypothesis was not tested probably due to technical difficulties. However, our mouse data imply that prestin plays an important role in separating supranuclear from subnuclear synapses early in OHC-MOC synapse formation (**Figure 5**). Although it is generally believed that the MOC pathway functions to protect against acoustic overstimulation by modulating OHC amplification, it is not clear what the role of these MOC-OHC synapses would be in prestin-KO mice where the hearing thresholds are already elevated (Liberman et al., 2002; Cheatham et al., 2007). Hence, further studies are required to elucidate other potential functions of the MOC system.

## Author Contributions

ST performed immunofluorescent experiments, analyzed the data, and prepared the figures. WS and BK performed TEM experiments. YZ and KH collected OHCs for RNA sequencing. MAC was involved in experimental design. ST, JZ, and MAC wrote the manuscript with input from all other authors.

## Conflict of Interest Statement

The authors declare that the research was conducted in the absence of any commercial or financial relationships that could be construed as a potential conflict of interest.
