# Supplementary material for: Prestin Contributes to Membrane Compartmentalization and Is Required for Normal Innervation of Outer Hair Cells
Source: Front Cell Neurosci. 2018 Jul 20;12:211. doi: 10.3389/fncel.2018.00211 (PMC6062617; doi:10.3389/fncel.2018.00211)
Supplement: Supplementary file 1 [file Presentation_1.pdf]

Supplementary Material

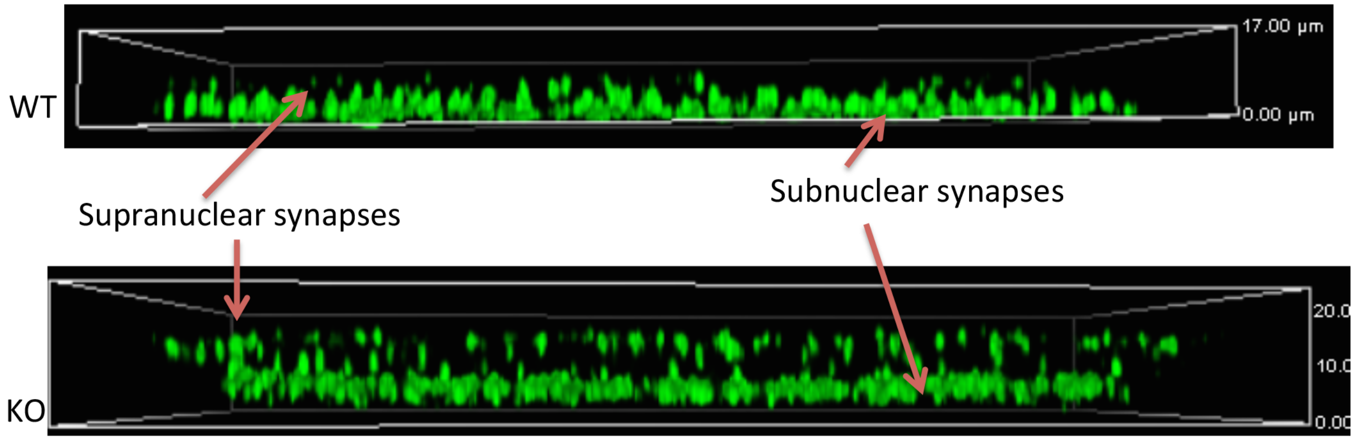

Figure S1. OHCs from prestin-KO mice are associated with more numerous and enlarged supranuclear synapses as compared to WT. Longitudinal view of the z-stack images from OHCs from WT (upper) and KO (lower). Compared to WT, MOC terminals in prestin-KO mice exhibited a noticeable two-layer pattern of synapses: one for subnuclear synapses similar to that of WT, another for supranuclear synapses. The two-layer pattern was not observed in WT and 499-prestin-KI-OHCs (data not show), which carry normal amounts of WT or mutant prestin protein. Two layers of terminals were observed in KO-OHCs (n=6) but not in WT-OHCs. Green: anti-synaptophysin for synaptic vesicles.

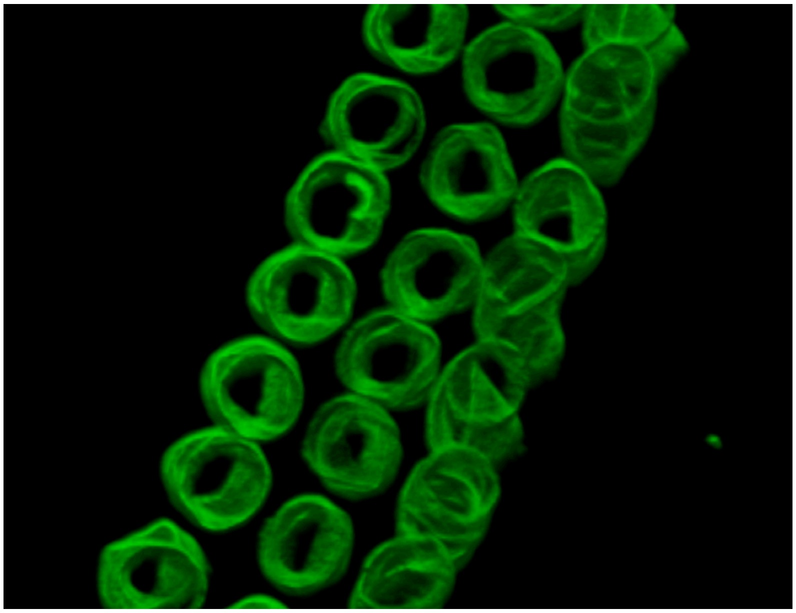

Figure S2. Prestin V499G/Y501H mutant is expressed at the LM of OHCs. OHCs from an adult 499-prestin-KI mouse were stained with anti-prestin (Green). The top view of a 3D reconstruction of a series of z-stack confocal images, ranging from the cuticular plate to the bottom of OHCs, is shown. OHCs appear as hollow cylinders.
